# Prevalence and influencing factors of hyperuricemia in middle-aged and older adults in the Yao minority area of China: a cross-sectional study

**DOI:** 10.1038/s41598-023-37274-y

**Published:** 2023-06-22

**Authors:** Xiao Lyu, Yuanxiao Du, Guoyu Liu, Tingyu Mai, You Li, Zhiyong Zhang, Chunhua Bei

**Affiliations:** 1 Department of Epidemiology and Health Statistics, School of Public Health, Guilin Medical University, Huan Cheng North 2nd Road 109, Guilin, 541004 Guangxi China; 2 Department of Environmental and Occupational Health, School of Public Health, Guilin Medical University, Huan Cheng North 2nd Road 109, Guilin, 541004 Guangxi China; 3 Guangxi Key Laboratory of Environmental Exposomics and Entire Lifecycle Heath, Guangxi Health Commission Key Laboratory of Entire Lifecycle Health and Care, School of Public Health, Guilin Medical University, Guilin, China

**Keywords:** Epidemiology, Endocrine system and metabolic diseases, Randomized controlled trials, Risk factors, Metabolic syndrome

## Abstract

Hyperuricemia (HUA) endangers human health, and its prevalence has increased rapidly in recent decades. The current study investigated HUA's prevalence and influencing factors in Gongcheng, southern China. A cross-sectional investigation was conducted; 2128 participants aged 30–93 years were included from 2018 to 2019. Univariate and multivariate logistic regression models were used to screen HUA variables. A Bayesian network model was constructed using the PC algorithm to evaluate the association between influencing factors and HUA. The prevalence of HUA was 15.6% (23.2% in men, 10.7% in women). After screening the variables using a logistic regression analysis model, fatty liver disease (FLD), dyslipidemia, abdominal obesity, creatinine (CREA), somatotype, bone mass, drinking, and physical activity level at work were included in the Bayesian network model. The model results showed that dyslipidemia, somatotype, CREA, and drinking were directly related to HUA. Bone mass and FLD were indirectly associated with HUA by affecting the somatotype. The prevalence of HUA in Gongcheng was high in China. The prevalence of HUA was related to somatotype, drinking, bone mass, physical activity level at work, and other metabolic diseases. A good diet and moderate exercise are recommended to maintain a healthy somatotype and reduce the prevalence rate of HUA.

## Introduction

Uric acid (UA) is the final product of purine metabolism, mainly derived from nucleic acids and other purine compounds decomposed by cell metabolism and purine in food^1^. UA level in human bodies is normally balanced; however, hyperuricemia (HUA) occurs as a result of increased accumulation or reduced excretion of UA^2^. HUA is the root cause of gout and UA nephropathy and an independent risk factor for hypertension, diabetes mellitus, and coronary heart disease^3^. Overall, the prevalence of HUA tends to be higher in developed countries than in developing countries. However, it has been noted that there is a persistent increase in the prevalence of HUA in many developing countries^4^.With the rapid development of China's economy, urbanization, aging population, and changes in traditional eating habits and lifestyles, the prevalence of HUA has increased from 11.1 (2015–2016) to 14.0% (2018–2019) in China^5^. According to data from the National Health and Nutrition Examination Survey (NHANES) 2007–2016, a nationally representative survey, the prevalence rates of HUA were 20.2% among men and 20.0% among women between 2015 and 2016 in the United States^6^. The prevalence of HUA increased in both men (19.7–25.0%) and women (20.5–24.1%) from 2006 to 2014 in Ireland^7^. Therefore, it has gradually developed into an important public health problem that seriously endangers public health and has caused huge health and economic burden to human beings.

Different regions have geographic characteristics, genetic factors, which predispose individuals to HUA. It was revealed that the pooled prevalence of HUA was significantly higher in the southern (25.5%) and southwestern (21.2%) regions of China than in the remaining regions^8^. The prevalence of HUA varies significantly across different ethnicities in China^9^. Among the Han population, the prevalence was found to be 17.9%^10^, while it was as high as 24.5% among the Zhuang community^11^. In contrast, the Mongolian ethnicity and Hui ethnicity had significantly lower HUA prevalence rates of 10.0% and 4.0%, respectively^12^. Interestingly, a recent survey conducted in Xinjiang highlighted that the Uighur community had one of the lowest HUA prevalence rates (4.6%), which was attributed to their low alcohol consumption^5^. Guangxi, located in southern China, has the largest population of ethnic minorities and a unique geographical environment, lifestyle, and national cultural characteristics. Gongcheng is a Yao autonomous county that is located in Guangxi, the southeast of Guilin, with a resident population of 360,000, and includes Yao, Han, and Zhuang people. Most Yao people live in deep mountains and are relatively isolated, making them different from the general population in terms of social culture and living habits. Therefore, this study conducted a cross-sectional survey to investigate the prevalence and influencing factors of HUA in adults aged ≥ 30 years among the Yao people of Guangxi to enrich the epidemiological study in the field of HUA among residents in rural minority areas of China.

## Materials and methods

### Study population

**Figure 1:**
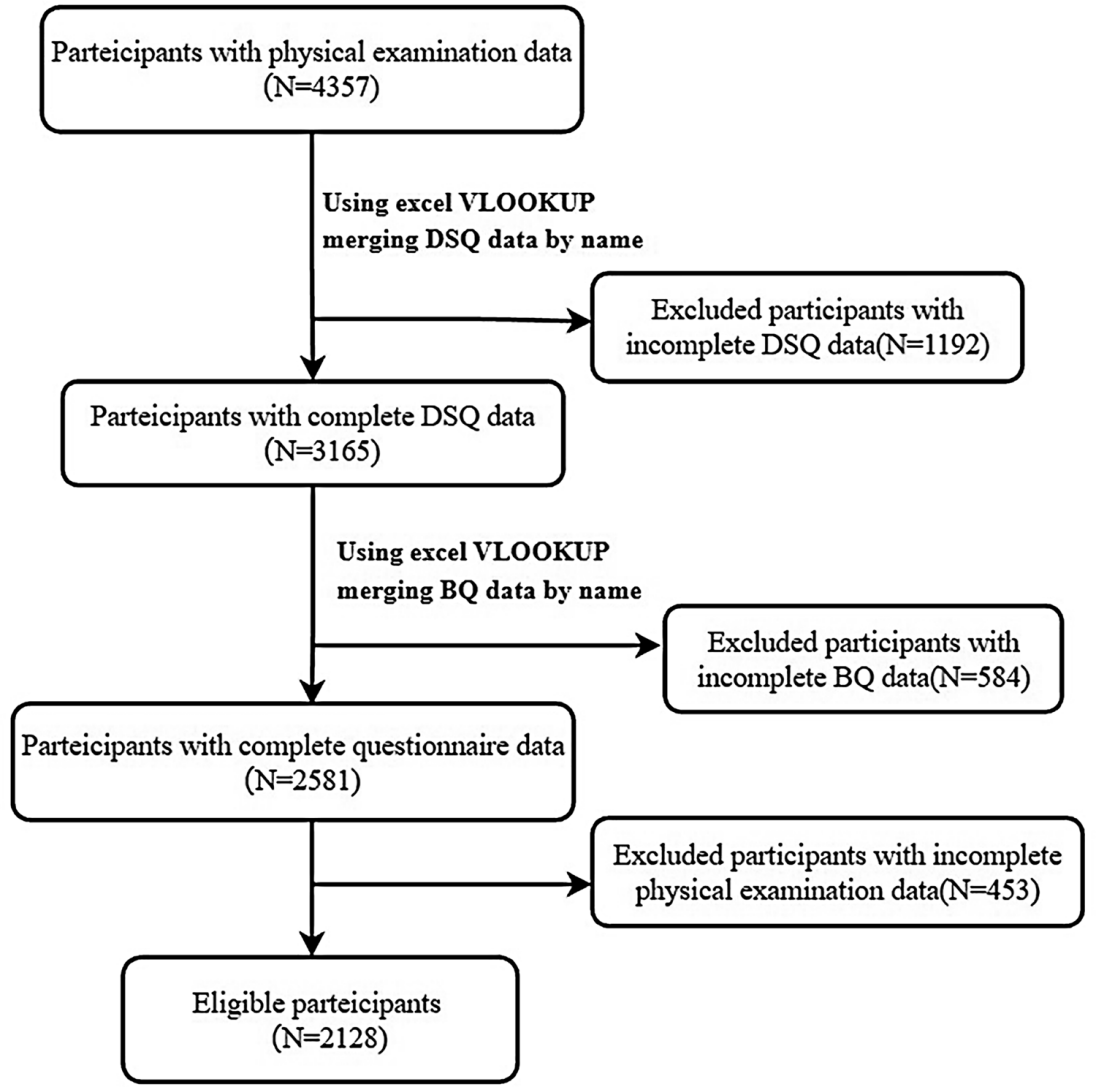
Flow chart of sample selection criteria: cross-sectional study. *DSQ* dietary assessment questionnaires, *BQ* baseline questionnaire.

The study was based on the Healthy Gongcheng Cohort Study. We aimed to understand the health status of people over 30 years old in Gongcheng to create a national health promotion county in Gongcheng. From December 2018 to December 2019, it was conducted a cross-sectional survey in Lianhua Town and Limu Town. All survey respondents participated voluntarily, and 4357 residents aged ≥ 30 years were screened for routine health. We conducted baseline and dietary assessment questionnaires a few months later and collected 3719 baseline and 3397 dietary assessment questionnaires. Residents aged ≤ 30 years, who had self-reported disability, mental disease malignant tumor, clinotherapy, or egresses, or who worked or lived away from home over half a year were excluded from further participation. After eliminating the questionnaires with missing information, incorrect information, or obvious errors*,* a total of 2128 participants were included in this study. The process was shown in Fig. 1. Participant’s personal information was de-identified and stored in protected files and locked cabinets. This cross-sectional study protocol was approved by the Ethics Committee of the Guilin Medical University (No. 20180702-3) and was in accordance with the Declaration of Helsinki (1964) and subsequent amendments. All participants signed an informed consent form.

### Data collection

Volunteers made up of medical students were recruited and trained for a week to develop the skills needed for the survey^13^. Physical examination items included height, weight, chest circumference, waist circumference, blood pressure, oxygen saturation, electrocardiogram, abdominal B ultrasound, chest anteroposterior film, bone mineral density, blood routine, blood biochemistry 13 items, urine routine and other examinations. The biochemical indexes were detected in the People's Hospital of Gongcheng Yao Autonomous County. Parallel samples were set for all routine physical examination indexes at testing time. The baseline questionnaire collected the demographic information of the participants, including gender, age, educational level, occupation, smoking, alcohol consumption, medical history, physical activity, sleep status, etc. The semi-quantitative food frequency questionnaire was used to collect the information of nutrients, drinking situation of Camellia oleifera, drinking frequency of green tea, daily oil intake, daily salt intake, etc.

### Definition

The HUA was defined as a serum UA level > 420 μmol/L in men and > 360 μmol/L in women^14^. Diabetes was defined as fasting plasma glucose (FPG) ≥ 126 mg/dL (7.0 mmol/L) or glycosylated hemoglobin (HbA1c) ≥ 48 mmol/mol (6.5%) or history of diabetes^15^. Fatty liver disease (FLD) was defined as per patient's self-reported medical history or abdominal B-ultrasound findings. The diagnostic criteria for dyslipidemia included serum total cholesterol (TC) ≥ 6.2 mmol/L, low-density lipoprotein (LDL) ≥ 4.1 mmol/L, high-density lipoprotein (HDL) < 1.0 mmol/L, and triglyceride (TG) ≥ 2.3 mmol/L^16^. Anemia was defined as hemoglobin less than 130 g/L for men and 120 g/L for women^17^. Hypoxia was defined as ambient air blood oxygen saturation < 90% by pulse oximetry at diagnosis^18^. The bone mass was defined according to World Health Organization criteria: normal if the T-score was > − 1.0; osteopenia if it was between − 1.0 and − 2.5; and osteoporosis if it was < − 2.5^19^. Body mass index (BMI) was calculated by weight (kg)/height (m^2^). Participants were classified as emaciation (BMI < 18.5 kg/m^2^), normal weight (BMI 18.5–24.9 kg/m^2^), overweight (BMI 25–29.9 kg/m^2^) and obese (BMI ≥ 30 kg/m^2^)^20^. Abdominal obesity was defined by waist circumference (WC) > 102 cm in men and WC > 88 cm in women^21^. Hypertension was defined as blood pressure ≥ 140/90 mm Hg or a history of treatment with antihypertensive medication^22^.

### Bayesian network model

The Bayesian network model is a kind of probabilistic graph model that combines probability theory and graph theory to reveal the probabilistic dependence relationship between variables (nodes), which is represented by directed acyclic graph (DAG)^23^. In the DAG, the nodes represent the random variables $U = \left\{ {X_{1} ,...,X_{n} } \right\}$, and the directed edges represent the direct probabilistic dependencies between the corresponding variables $X_{1}$ and $X_{j}$.If there is an arc from $X_{1}$ to $X_{j}$,then we can informally say that $X_{1}$ causes $X_{j}$, so $X_{1}$ and $X_{j}$ are often referred to as parent and child, respectively is used to quantitatively describe the strength of the relationship between a random variable and its parent. A Bayesian network is simply a representation of the joint probability distribution of a set of random variables $X = \left\{ {X_{1} ,...,X_{n} } \right\}$, so the probability expression can be obtained:

$$P(X_{1} ,...X_{n} ) = \prod\limits_{i = 1}^{n} {|P(X_{i} |X_{1} ,X_{2,...,} X_{i - 1} )} = \prod\limits_{i = 1}^{n} {|P(X_{i} |\pi (X_{i} )} )$$

where $\pi (X_{i} )$ represents the set of parent nodes of node $X_{i}$, $\pi (X_{i} ) \subseteq \left\{ {X_{1} ,...,X_{i - 1} } \right\}$^24^. A conditional probability table (CPT) can describe the association strength between variables by constructing a DAG to reveal the potential relationship between influencing factors^24^. This can intuitively clarify the complex internal regulation relationship between diseases and their related factors to make up for some shortcomings of the logistic regression analysis model^25^.

### Bayesian network learning algorithms

The learning of Bayesian network includes structure learning and parameter learning^26^. Structure learning is the process of constructing and determining the most suitable topological structure of Bayesian network from data, and its emphasis is to reveal the complex network relations among variables. Parameter learning is to determine the parameters of the model, the conditional probabilities and transition probabilities of the variables in the network, when the structure of the model is known. In this paper, the PC algorithm in GeNle4.0 software is used for structure learning, and EM (expectation–maximization) algorithm is used for parameter learning. The PC structure learning algorithm is one of the earliest and the most popular algorithms, it uses independences observed in data (established by means of classical independence tests) to infer the structure that has generated them.

### Statistical analysis

**Figure 2:**
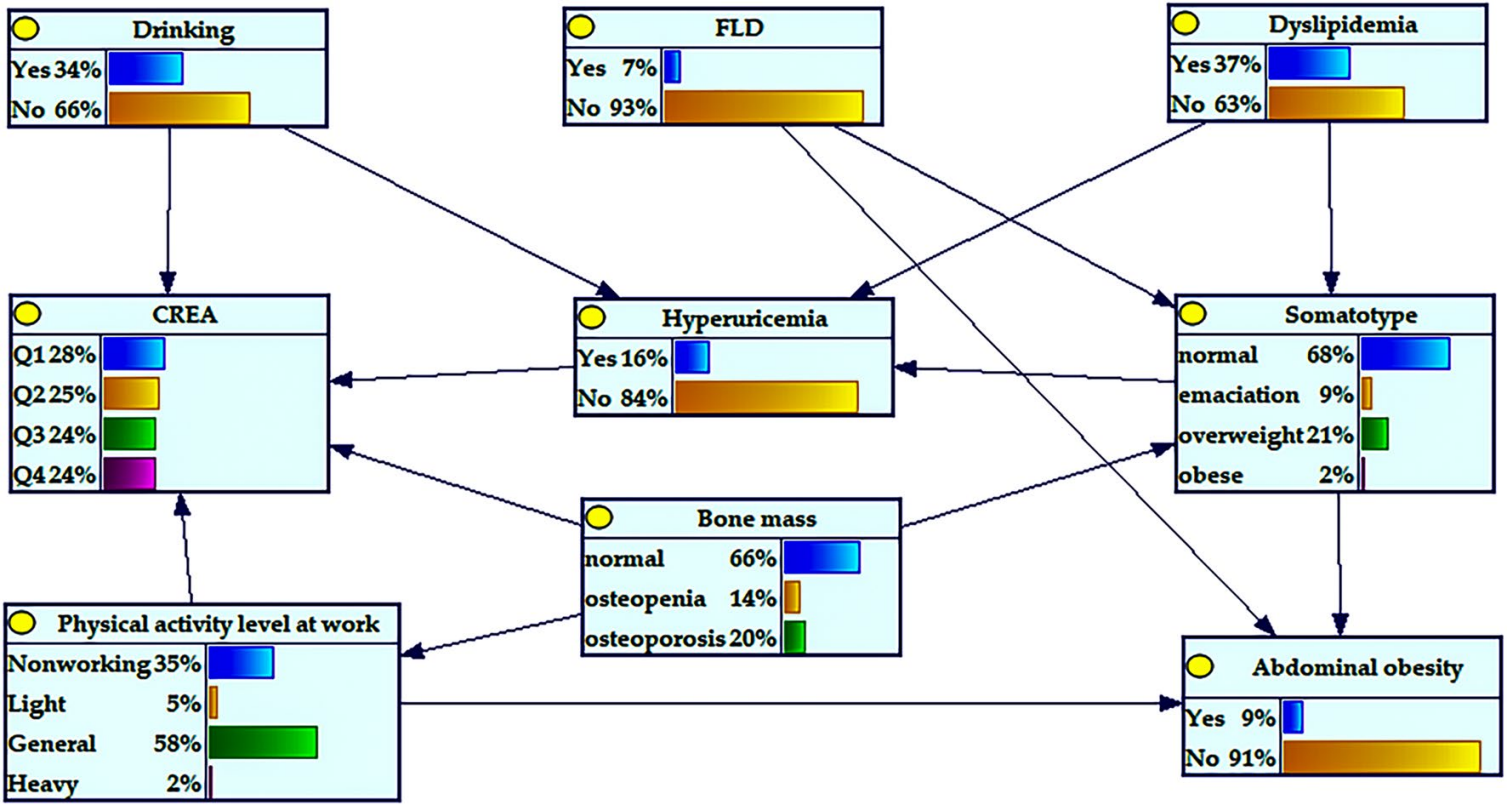
Bayesian network model of hyperuricemia based on EM algorithm and its prior probability. *FLD* fatty liver disease, *CREA* creatinine.

**Figure 3:**
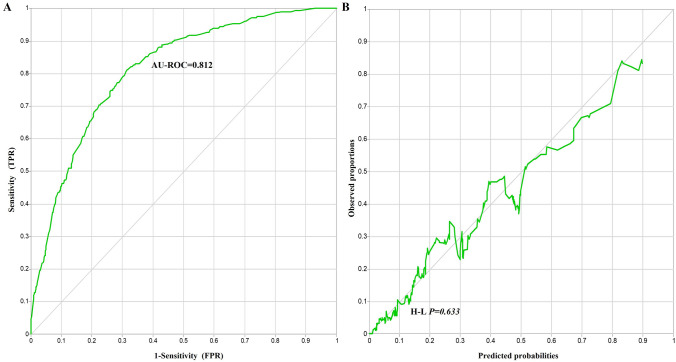
Prediction differentiation and accuracy of Bayesian network model in HUA. (**A**) The ROC curve for HUA. (**B**) The Calibration curve for HUA. *TPR* true positive rate, *FPR* false positive rate, *H–L* Hosmer–Lemeshow.

All data were collated using Microsoft Excel 2021. The normality of continuous variables was tested by using the Kolmogorov–Smirnov test. Data of all continuous variables that did not obey normality were presented as median and IQR. Categorical variables were described in percentages. Univariate analysis of categorical variables between HUA and non-HUA groups was performed using the Chi-square test and between creatinine (CREA) using the Mann–Whitney U test. The variables with statistical significance in univariate analysis were analyzed by binary logistic multivariate regression. The above statistical analysis was performed by SPSS28.0 software (IBM, Chicago, IL, USA) and 2-tailed *P* values < 0.05 were considered significant. The visualization of Fig. 1 was performed using QuickDraw software and the forest plot was visualized using GraphPad Prism 9.3.0 software shown in Fig. 2. The PC algorithm and EM algorithm of GeNle 4.0 Academic software were used to learn the structure and parameters of Bayesian network model, respectively. Bayesian network model graphs and CPTs were constructed using GeNle 4.0 Academic software as shown in Fig. 3.

### Institutional review board statement

The study was conducted in accordance with the Declaration of Helsinki, and approved by Ethics Committee of Guilin Medical University (No. 20180702-3).

### Informed consent statement

Informed consent was obtained from all participants involved in the study.

## Results

### Basic demographic characteristics

A total of 2128 participants were included in this cross-sectional study, of whom 826 (33.3%) were men, 1302 (61.2%) were women, ranging in age from 30 to 93 years, with a mean age of 57.7 ± 12.0 years. The overall prevalence of HUA in this population was 15.6%, 23.2% in men and 10.7% in women, respectively (Table 1).

**Table 1:** Univariate analysis of the prevalence of hyperuricemia in the cross-sectional study.

Characteristics	Total (n = 2128)	Hyperuricemia status (%)		*χ2/z*	*P* value
		Non-hyperuricemia (n = 1797)	Hyperuricemia (n = 331)		
Sex, n (%)				60.781	< 0.001*
Men	826 (33.3)	64 (76.8)	192 (23.2)		
Women	1302 (61.2)	1163 (89.3)	139 (10.7)		
Age (y), n (%)				5.601	0.061
30–44	291 (13.7)	259 (89.0)	32 (11.0)		
45–59	762 (35.8)	634 (83.2)	128 (16.8)		
≥ 60	1075 (50.5)	904 (84.1)	171 (15.9)		
Nation, n (%)				2.221	0.136
Yao	1587 (74.6)	1351 (85.1)	236 (14.9)		
Other	541 (25.4)	446 (82.4)	95 (17.6)		
Education level, n (%)				8.702	0.003*
< Middle school	1410 (66.3)	1214 (86.1)	196 (13.9)		
≥ Middle school	718 (33.7)	583 (81.2)	135 (18.8)		
Occupation, n (%)				3.781	0.052
Farmer	1965 (92.3)	1668 (84.9)	297 (15.1)		
Other	163 (7.7)	129 (79.1)	34 (20.9)		
Household income (RMB/year), n (%)				0.83	0.362
< 5000	580 (27.3)	483 (83.3)	97 (16.7)		
≥ 5000	1548 (72.7)	1314 (84.9)	234 (15.1)		
Operation, n (%)				0.399	0.527
Yes	1104 (51.9)	927 (84.0)	177 (16.0)		
No	1024 (48.1)	870 (85.0)	154 (15.0)		
Medical insurance, n (%)				1.686	0.194
Yes	2073 (97.4)	1754 (84.6)	319 (15.4)		
No	55 (2.6)	43 (78.1)	12 (21.9)		
Smoking, n (%)				27.653	< 0.001*
Yes	397 (18.7)	301 (75.8)	96 (24.2)		
No	1731 (81.3)	1496 (86.4)	235 (13.6)		
Drinking, n (%)				18.3	< 0.001*
Yes	727 (34.2)	580 (79.8)	147 (20.2)		
No	1401 (65.8)	1217 (86.9)	184 (13.1)		
Daily oil intake(g), n (%)				2.189	0.139
< 25	1076 (50.6)	921 (85.6)	155 (14.4)		
≥ 25	1052 (49.4)	876 (83.3)	176 (16.7)		
Daily salt intake(g), n (%)				3.493	0.062
< 5	1837 (86.3)	1562 (85.0)	275 (15.0)		
≥ 5	291 (13.7)	235 (80.8)	56 (19.2)		
Oil tea, n (%)				6.235	0.013*
Yes	2040 (95.9)	1731 (84.9)	309 (15.1)		
No	88 (4.1)	66 (75.0)	22 (25.0)		
Green tea, n (%)				1.01	0.315
Yes	771 (36.2)	643 (83.4)	128 (16.6)		
No	1357 (63.8)	1154 (85.0)	203 (15.0)		
Physical activity level at work, n (%)				11.789	0.008*
Non-working	741 (34.8)	600 (81.0)	141 (19.0)		
Light	106 (5.0)	88 (83.0)	18 (17.0)		
General	1232 (57.9)	1065 (86.4)	167 (13.6)		
Heavy	49 (2.3)	44 (89.8)	5 (10.2)		
Walking time, n (%)				3.274	0.195
≤ 2 h/day	1046 (49.2)	871 (83.3)	175 (16.7)		
2–4 h/day	261 (12.3)	218 (83.5)	43 (16.5)		
> 4 h/day	821 (38.6)	708 (86.2)	113 (13.8)		
Working time, n (%)					
≤ 8 h/day	2005 (94.2)	1688 (84.2)	317 (15.8)	1.73	0.188
> 8 h/day	123 (5.8)	109 (88.6)	14 (11.4)		
Weekly working days, n (%)				15.402	< 0.001*
≤ 5 days	654 (30.7)	522 (79.8)	132 (20.2)		
> 5 days	1474 (69.3)	1275 (86.5)	199 (13.5)		
Diabetes, n (%)				17.648	< 0.001*
Yes	222 (10.4)	166 (74.8)	56 (25.2)		
No	1906 (89.6)	1631 (85.6)	275 (14.4)		
FLD, n (%)				43.484	< 0.001*
Yes	156 (7.3)	103 (66.0)	53 (34.0)		
No	1972 (92.7)	1694 (85.9)	278 (14.1)		
Dyslipidemia, n (%)				79.238	< 0.001*
Yes	797 (37.5)	601 (75.4)	196 (24.6)		
No	1331 (62.5)	1196 (89.9)	135 (10.1)		
Hypertension, n (%)				2.769	0.096
Yes	889 (41.8)	737 (82.9)	152 (17.1)		
No	1239 (58.2)	1060 (85.6)	179 (14.4)		
Anemia, n (%)				0.086	0.77
Yes	304 (14.3)	255 (83.9)	49 (16.1)		
No	1824 (85.7)	1542 (84.5)	282 (15.5)		
Hypoxia, n (%)				5.812	0.016*
Yes	91 (4.3)	85 (93.4)	6 (6.6)		
No	2037 (95.7)	1712 (84.0)	325 (16.0)		
Bone mass, n (%)				8.141	0.017*
Normal	1412 (66.4)	1171 (82.9)	241 (17.1)		
Osteopenia	297 (14.0)	264 (88.9)	33 (11.1)		
Osteoporosis	419 (19.7)	362 (86.4)	57 (13.6)		
Somatotype, n (%)				72.901	< 0.001*
Normal	1450 (68.1)	1261 (87.0)	189 (13.0)		
Emaciation	183 (8.6)	171 (93.4)	12 (6.6)		
Overweight	446 (21.0)	337 (75.6)	109 (24.4)		
Obese	49 (2.3)	28 (57.1)	21 (42.9)		
Abdominal obesity, n (%)				25.673	< 0.001*
Yes	182 (8.6)	130 (71.4)	52 (28.6)		
No	1946 (91.4)	1667 (85.7)	279 (14.3)		
CREA, M (P25, P75)	67.0 (58.0,79.0)	65.0 (57.0,76.0)	81.0 (69.0,96.0)	13.789	< 0.001*

*FLD* fatty liver disease CREA, creatinine.

*p value < 0.05.

### Univariate analysis

The univariate analysis of the questionnaire data and physical examination data on the prevalence of HUA were shown in Table 1. The prevalence of HUA in men (826, 23.2%) was significantly higher than that of women (1302, 10.7%). Similarly, the educational level (≥ middle school) smoking, drinking, hypoxia, abdominal obesity and weekly working days (≤ 5 days) were risk factors for HUA. In addition, both oil tea and bone mass (osteopenia, osteoporosis) were protective factors against HUA in public. Non-working and obese showed the highest prevalence among the four physical activity level at work groups and somatotype group, respectively (*P* < 0.05). Interestingly, there were no significant differences between the HUA group and the non-HUA group in age, nation, occupation, household income, operation, medical insurance, daily oil/salt intake, green tea, walking time, and working time. Moreover, it was showed significant differences between the HUA group and the non-HUA group in other metabolic diseases (diabetes, FLD, dyslipidemia) and CREA level (*P* < 0.05). The occurrence of HUA was not related to hypertension and anemia (Table 1).

### Multivariate logistic regression analysis

To simplify the structure of the later Bayesian network model, 15 variables with *P* < 0.05 in univariate analysis were included in the binary logistic multivariate regression analysis model. The relevant factors affecting the occurrence of HUA were screened with *α*_in_ = 0.05 and* α*_out_ = 0.10, as shown in Table 2. The logistic regression analysis results showed that alcohol consumption, physical activity level at work, FLD, dyslipidemia, bone mass, abdominal obesity, somatotype, and CREA finally entered the model. Drinking, FLD, dyslipidemia, abdominal obesity, somatotype (overweight, obesity), and CREA were risk factors for HUA. In addition, physical activity level at work and bone mass (osteopenia) were protective factors against HUA.

**Table 2:** Multivariate logistic analysis of the prevalence of hyperuricemia in the cross-sectional study.

Characteristics	*β*	*OR (95%CI)*	*P value*
Sex			
Men		1.000	
Women	0.296	1.345 (0.877, 2.063)	0.174
Education level			
< middle school		1.000	
≥ middle school	0.166	1.204 (0.917, 1.581)	0.255
Smoking			
No		1.000	
Yes	0.267	1.306 (0.914, 1.867)	0.142
Drinking			
No		1.000	
Yes	0.457	1.579 (1.177, 2.118)	0.002*
Weekly workdays			
≤ 5		1.000	
> 5	− 0.103	0.902 (0.661, 1.230)	0.514
Oil tea			
No		1.000	
Yes	− 0.434	0.648 (0.366, 1.148)	0.137
Physical activity level at work			0.028*
Non-working		1.000	
Light	− 0.001	0.999 (0.532, 1.873)	0.997
General	− 0.334	0.716 (0.517, 0.991)	0.044
Heavy	− 1.361	0.256 (0.088, 0.745)	0.012
Diabetes			
No		1.000	
Yes	0.352	1.422 (0.967, 2.091)	0.073
FLD			
No		1.000	
Yes	0.794	2.212 (1.416, 3.456)	< 0.001*
Dyslipidemia			
No		1.000	
Yes	0.931	2.536 (1.942, 3.312)	< 0.001*
Bone mass			0.045*
Normal		1.000	
Osteopenia	− 0.446	0.640 (0.412, 0.994)	0.047
Osteoporosis	0.196	1.217 (0.828, 1.788)	0.317
Hypoxia			
No		1.000	
Yes	− 0.857	0.425 (0.170, 1.058)	0.066
Abdominal obesity			
No		1.000	
Yes	0.523	1.687 (1.039, 2.740)	0.035*
Somatotype			0.005*
Normal		1.000	
Emaciation	− 0.362	0.696 (0.364, 1.334)	0.275
Overweight	0.474	1.607 (1.165, 2.215)	0.004
Obese	0.879	2.409 (1.137, 5.104)	0.022
CREA			< 0.001*
Q1		1.000	
Q2	0.769	2.157 (1.314, 3.543)	0.002
Q3	1.285	3.615 (2.202, 5.937)	< 0.001
Q4	2.506	12.259 (7.325, 20.516)	< 0.001
* β0*	− 3.528	0.029	< 0.001*

*FLD* fatty liver disease, *CREA* creatinine. Q1 through Q4 represent the first and last IQR for creatinine, respectively.

*p value < 0.05.

In contrast, drinking was associated with a 57.9% (*OR* = 1.579, 95% *CI* 1.177–2.118, *P* = 0.002) higher odds for HUA. Similarly, the risk of HUA was 28.4% (*OR* = 0.716, 95% *CI* 0.517–0.991, *P* = 0.044) and 74.4% *(OR* = 0.256, 95% *CI* 0.088–0.745, *P* = 0.012) lower in the population with general and heavy physical activity levels at work, respectively. Moreover, the odds of HUA in patients with FLD and dyslipidemia were 1.212 times (*OR* = 2.212, 95% *CI* 1.416–3.456, *P* < 0.001) and 1.536 times (*OR* = 2.536, 95% *CI* 1.942–3.312,* P* < 0.001) higher than those in patients with non-FLD and normal lipid levels, respectively. Interestingly, the risk of HUA was 0.36 times (*OR* = 0.640, 95% *CI* 0.412–0.994, *P* = 0.047) lower in the osteopenia group than in the control group. In addition, compared with the normal weight group, the risk of HUA increased by 60.7% (*OR* = 1.607, 95% *CI* 1.165–2.215, *P* = 0.004) and 1.409 times (*OR* = 2.409, 95% *CI* 1.314–3.543,* P* = 0.022) in the overweight and obese groups, respectively. According to the IQR of the CREA, the participants were divided into groups Q1–Q4. Compared to the lowest quartile of CREA, those in groups Q2, Q3, and Q4 had a significant association with a higher rate of HUA (*OR* = 2.157, 95% *CI* 1.314–3.543; *OR* = 3.615, 95% *CI* 2.202–5.937; and *OR* = 12.259, 95% *CI* 7.325–20.516, respectively; all *P* values are less than 0.05) (Table 2).

### Bayesian network model of HUA

According to the eight variables screened from the logistic regression analysis model, the Bayesian network model of related factors of HUA was further constructed using the EM algorithm in GeNle4.0 software. As shown in Fig. 2, a HUA Bayesian network model containing nine nodes and 14 directed edges was constructed. The results showed that drinking, dyslipidemia, somatotype, and CREA were directly related to HUA, in which drinking, dyslipidemia, and somatotype were the father nodes of HUA, and the child node of HUA was CREA. Bone mass and FLD were indirectly related to HUA by affecting somatotype, suggesting that somatotype was the intermediate variable between bone mass and FLD, affecting the occurrence of HUA, as shown in Fig. 2.

The calibration curve for predicting incidence and observed proportions closely followed the line of y = x. Additionally, the Hosmer–Lemeshow goodness of fit test yielded a *P* value of 0.633, greater than the significance level of 0.05. These results indicated that the Bayesian network model was well calibrated, as depicted in Fig. 3A. Moreover, the area under the curve (AUC) of the receiver operating characteristic (ROC) was found to be 0.812, which was greater than the cutoff value of 0.750. This suggested that the variables in the Bayesian network model had good discriminatory ability, as illustrated in Fig. 3B.

### Risk reasoning of HUA

The Bayesian network model can infer the probability of unknown nodes according to the state of known nodes to determine the risk of HUA. The risk of HUA was the lowest (0.036) in patients with dyslipidemia and emaciation and in those who did not drink. The risk of HUA was highest when dyslipidemia, obesity, and drinking were present (0.773). The risk of developing HUA increased significantly with the change in somatotype from emaciation to obesity (Table 3).

**Table 3:** Conditional probability table of hyperuricemia nodes in Gongcheng, Guangxi.

Dyslipidemia	Somatotype	Drinking	Risk of hyperuricemia
Yes	Normal	Yes	0.237
Yes	Normal	No	0.217
Yes	Emaciation	Yes	0.045
Yes	Emaciation	No	0.036
Yes	Overweight	Yes	0.454
Yes	Overweight	No	0.215
Yes	Obese	Yes	0.773
Yes	Obese	No	0.531
No	Normal	Yes	0.123
No	Normal	No	0.060
No	Emaciation	Yes	0.074
No	Emaciation	No	0.095
No	Overweight	Yes	0.238
No	Overweight	No	0.163
No	Obese	Yes	0.239
No	Obese	No	0.167

*FLD* fatty liver disease.

## Discussion

HUA has become a common metabolic disease, which is affected by economic development, environment, diet, race, heredity and other factors^27^ The prevalence of HUA in China increased from 8.4^8^ to 14.0%^5^ during 2001–2019. In this cross-sectional study of adults from Gongcheng Yao Autonomous County, the prevalence rate of HUA was 15.6% (23.2% for men, 10.7% for women), corresponding to an estimated 38.3 thousand adults with HUA, which was higher than that reported in other neighboring Asian countries such as Japan (13.4%)^28^ and Korea (11.4%)^29^. Moreover, the HUA prevalence in Gongcheng men (23.2%) was significantly higher than that found in some developed countries such as the USA^6^ (20.2%) and Australia^30^ (16.6%). The HUA prevalence in China is similar to that in developed countries.We hypothesized that the increased prevalence rates of HUA in Gongcheng may be related to China's rapid economic development and westernization of dietary habits in recent years^31^.

It has been observed that HUA is more prevalent in individuals of Zhuang ethnicity compared to those of Han descent, with a reported prevalence of 24.5% in Guangxi in 2018–2019^11^. Furthermore, recent research has identified Zhuang descent as a risk factor for HUA^5^. However, in our study, the prevalence of HUA in Yao individuals was found to be lower (11.1%) than that in Han and Zhuang. Interestingly, even though the diet of Tibetan people is usually rich in meat, fat, and alcohol, the prevalence of HUA (2.05%) was still lower than that of Han (17.9%)^32^. Additionally, despite the Inner Mongolia Mongolian residents' diet primarily comprising meat and dairy products, the prevalence of HUA (10.0%) was also found to be lower in Mongo than in Han people^12^. Major et al.^33^ reported that genetic variants may play a greater role in hyperuricaemia in the general population compared with dietary exposure, which could explain the varying prevalence found in these studies. However, further research to confirm these findings is needed.

Previous studies reported that the prevalence of HUA in men was higher than that in women^34,35^. In the current study, our results demonstrated sex differences in the prevalence of HUA, which was markedly higher in men (23.2%) than in women (10.7%). The results of the univariate analysis were significant. Such sex differences may be related to the higher estrogen level in women, which benefits UA excretion. In comparison, higher androgen level in men promotes renal reabsorption of UA and inhibits UA excretion^30,36^, owing to the lifestyle of men consisting of drinking and a high-fat and high-purine diet. However, the effect of sex was not significant after multivariate analysis, which may be owing to the small proportion of sex and other factors affecting HUA. This study further demonstrated that FLD, dyslipidemia, drinking, abdominal obesity, somatotype (overweight, obese), and CREA levels were all risk factors for HUA, which was consistent with the studies of other regions in China and with other ethnic groups^37–40^. Physical activity level at work and osteopenia were protective factors against HUA^5,41^. We speculated that the plasma volume increases with the increase in glomerular filtration rate and extracellular fluid volume during long-term moderate exercise, and the improvement in renal plasma flow would promote the secretion and excretion of UA^42^. We realize that the potential influencing factors analyzed in this study were limited; the investigation of more factors is warranted in future studies.

In addition, we found that the Bayesian network model diagram further demonstrated the complex network connection among the various influencing factors of HUA, among which dyslipidemia, somatotype, and drinking were directly related to HUA. Risk inference by the Bayesian network model showed that the risk of HUA was the highest in people with dyslipidemia and obesity and in those who drank. This might be because the lipotoxicity in dyslipidemia affects the function of islet β cells, increases the levels of free fatty acids, and promotes the occurrence of β-oxidation of free fatty acids; this enhances the activity of NADPH, promotes the synthesis of UA, and causes HUA^39^. The possible causes are visceral fat accumulation^36^, endocrine system disorder, androgen and ACTH level decrease, and UA excretion inhibition, which might lead to HUA complications^43^. The synthesis and metabolism of lactic acid would be accelerated during alcohol metabolism in those who consume alcohol, and lactic acid competitively inhibits UA secretion from renal tubules, activates the ion exchange function of the human urate anion exchanger, inhibits UA excretory function of the kidney, and stimulates UA reabsorption in proximal tubules^44,45^. In addition, people often consume purine-rich foods during drinking, which would further increase UA content and cause HUA^42^. At the same time, compared with the non-HUA group, the CREA level in the HUA group was significantly higher. Relevant studies^46,47^ showed that the serum CREA level was the most commonly used renal function indicator, which might lead to chronic kidney disease (CKD). HUA can indirectly affect renal function by affecting the serum CREA level. It is worth noting that emaciated people with normal blood lipid levels had a lower risk of developing HUA. This may be because being emaciated and dyslipidemic may jointly contribute to a higher risk of developing HUA; however, further studies are needed in this regard. The results also suggested that bone mass and FLD were associated with the somatotype and that somatotype was directly associated with HUA. We assumed that might be because patients with osteopenia and FLD are mostly overweight and obese, thus promoting insulin resistance, reducing renal UA excretion, and leading to HUA^48^.

Our study had several strengths. First, to our knowledge, this study is the first to investigate the prevalence of HUA in a large sample of Yao individuals, thereby providing valuable insights into the prevalence of this condition across different ethnic groups in China. Second, compared with previous studies on HUA, which used logistic and Cox regression models to describe several independent factors of HUA, the Bayesian network model could reveal how the factors were related to each other and affected the occurrence of HUA through the form of a probabilistic graphical model. This helped discover the potential influencing factors of the disease and provided new clues for further research. Third, this study was based on a survey of the natural population in ethnic minority areas. In addition to the physical examination data, a large amount of detailed questionnaire data was combined. The survey results had important practical significance for determining the prevalence of HUA in ethnic minority areas and specifying corresponding prevention and control strategies. However, some limitations of this study should be noted. First, most of the participants in this study were located in Gongcheng Yao Autonomous County, which might not be sufficient to represent the overall prevalence of HUA in Guangxi ethnic minorities. Second, as a cross-sectional study, the causal relationship between HUA and risk factors could not be determined, and further prospective studies are needed to demonstrate this.

## Conclusions

In conclusion, this study has shown that the prevalence rates of HUA among adults in Gongcheng Yao Autonomous County during 2018–2019 were much higher than those reported in previous studies of the Chinese population and even higher than those found in some developed countries. The Bayesian network model can further supplement the complex network relationship among variables that cannot be displayed by the former and can more intuitively reveal the network relationship between diseases and related factors. This further suggests that the prevalence of HUA is influenced by a few factors, including somatotype, drinking, and other complicating metabolic diseases (such as FLD, dyslipidemia, and CKD). Interestingly, bone mass and physical activity level at work were independent protective factors against HUA. Thus, it is suggested to carry out health education for the population, guide the formation of a healthy lifestyle, and improve the blood UA level through good diet, alcohol restriction, adherence to moderate exercise, and maintaining a healthy and ideal somatotype to reduce the prevalence of HUA in the future.

## Data Availability

The datasets used or analyzed during the current study are available from the corresponding author on reasonable request.

## Abbreviations

HUA: Hyperuricemia
UA: Uric acid
DAG: Directed acyclic graph
CPT: Conditional probability table
DSQ: Dietary assessment questionnaires
BQ: Baseline questionnaire
EM: Expectation–maximization
BMI: Body mass Index
FLD: Fatty liver disease
CREA: Creatinine
OR: Odds ratio
95%CI: 95% Confident limit
CKD: Chronic kidney disease
